# Supplementary material for: SNPs in Sheep: Characterization of Lithuanian Sheep Populations
Source: Animals (Basel). 2021 Sep 9;11(9):2651. doi: 10.3390/ani11092651 (PMC8467540; doi:10.3390/ani11092651)
Supplement: Supplementary file 1 [file animals-11-02651-s001.zip › animals-1338741-supplementary.pdf]

Supplementary Materials

SNPs in Sheep: Characterization of Lithuanian Sheep Populations

Ruta Sveistiene [1,*] and Miika Tapio [2]

[1] Animal Science Institute, Lithuanian University of Health Sciences, 82317 Baisogala, Lithuania
[2] Natural Resources Institute Finland, 00790 Helsinki, Finland; miika.tapio@luke.fi
* Correspondence: ruta.sveistiene@lsmuni.lt; Tel.: +370-61214095

Table S1. Pairwise *Fst* estimates between populations based on 9583 SNP markers.

	LCWO	LBFA	LSKU	DHOR	SBFA	WILT	FINN	BHMU	EFBR	GTEX	ONSS	BOBE	SBBM	SMIR	SWAL
Lithuanian Coarsewooled															
Lithuanian Blackface	0.094														
Lithuanian Skudden	0.130	0.078													
Dorset Horn	0.154	0.096	0.130												
Scottish Blackface	0.098	0.044	0.079	0.094											
Wiltshire	0.175	0.107	0.151	0.162	0.108										
Finnsheep	0.093	0.045	0.074	0.098	0.046	0.114									
Black Headed Mutton	0.109	0.040	0.091	0.107	0.056	0.120	0.059								
East Friesian Brown	0.140	0.084	0.115	0.138	0.085	0.158	0.079	0.096							
German Texel	0.103	0.046	0.085	0.102	0.052	0.109	0.051	0.056	0.080						
Old Norwegian Spaelsau	0.097	0.047	0.076	0.096	0.043	0.112	0.035	0.059	0.083	0.051					
Bundner Oberlander	0.102	0.054	0.083	0.103	0.053	0.123	0.055	0.066	0.094	0.060	0.054				
Swiss Black-Brown Mountain	0.101	0.046	0.081	0.100	0.050	0.118	0.051	0.057	0.090	0.053	0.053	0.053			
Swiss Mirror	0.103	0.051	0.085	0.103	0.052	0.118	0.053	0.062	0.093	0.054	0.055	0.051	0.052		
Swiss White Alpine	0.102	0.047	0.085	0.102	0.053	0.111	0.053	0.059	0.092	0.048	0.055	0.049	0.051	0.039	
Valais Blacknose	0.125	0.075	0.103	0.124	0.073	0.146	0.074	0.087	0.115	0.082	0.073	0.072	0.071	0.076	0.079

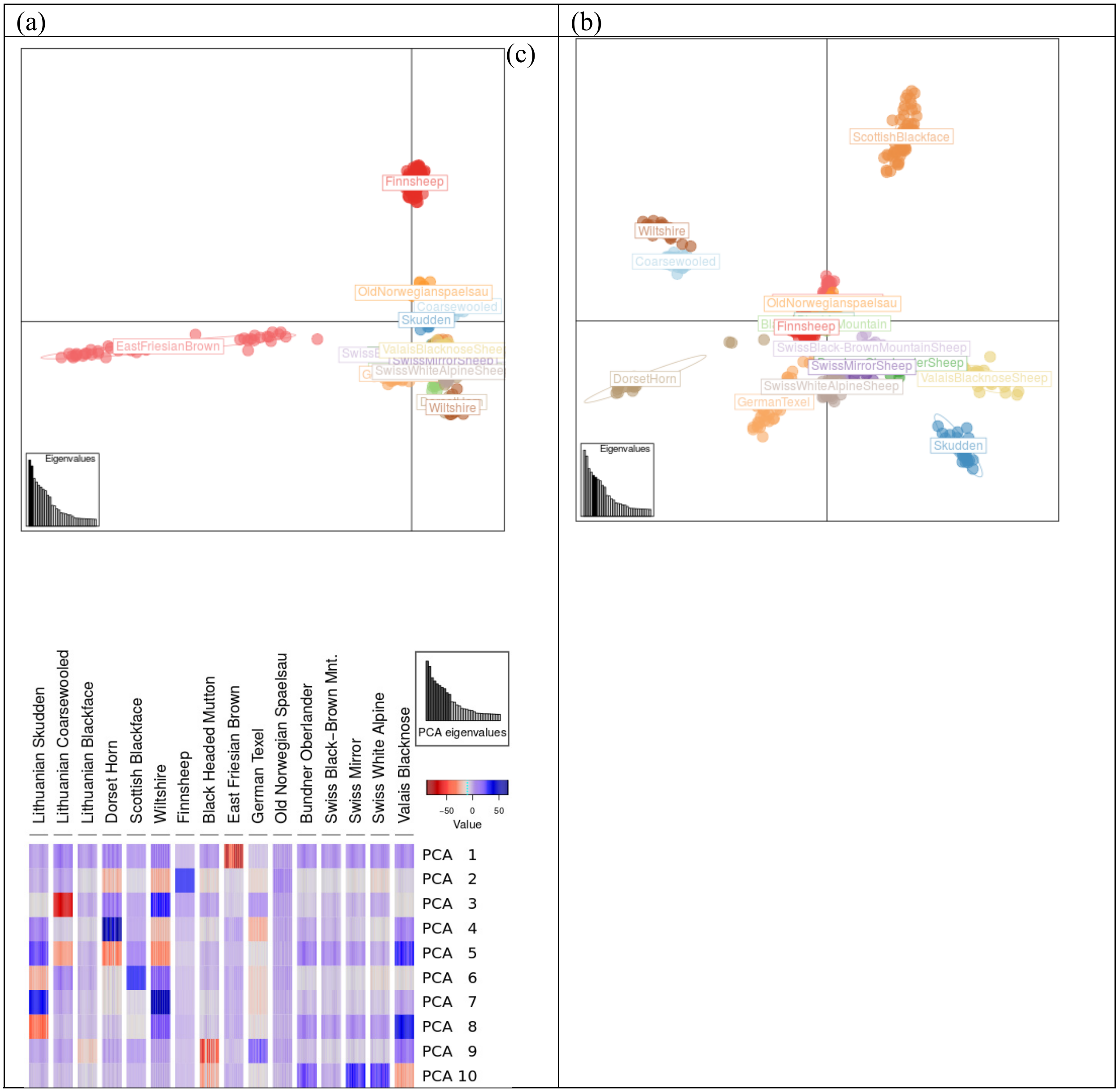

Figure S1. Plot of the two first principal components (a), plot of fifth and sixth component (b) and heat-map for ten first principal components (c) for the Lithuanian and reference populations. In (c) similar colour on a row indicate similar component values for the respective component. In the plots, the small screen diagrams indicate the plotted components (darkened).

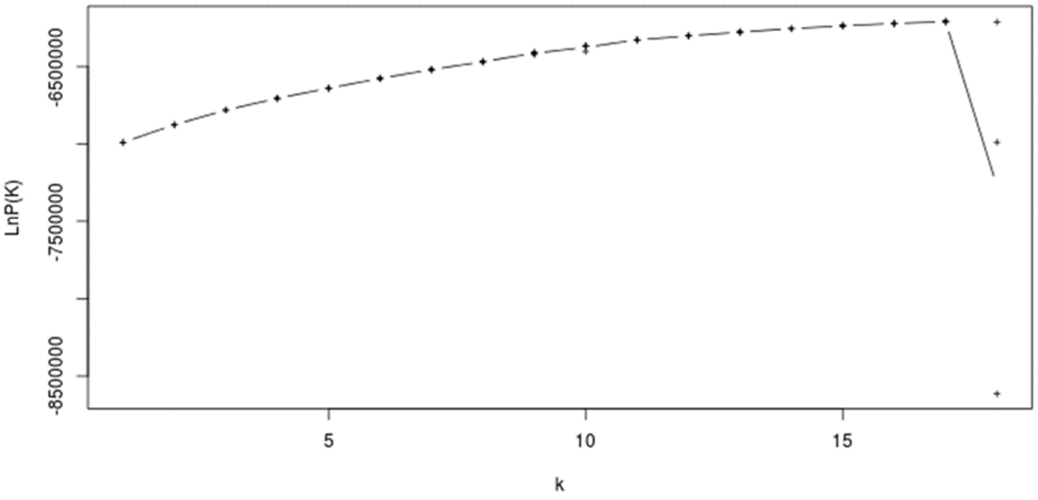

Figure S2. LnPD (K) plotted for all values of *k*. The crosses show individual estimates and line combines their mean values. At k = 18 the iterations do not converge and only one repeat found a good solution with the used chain lengths.

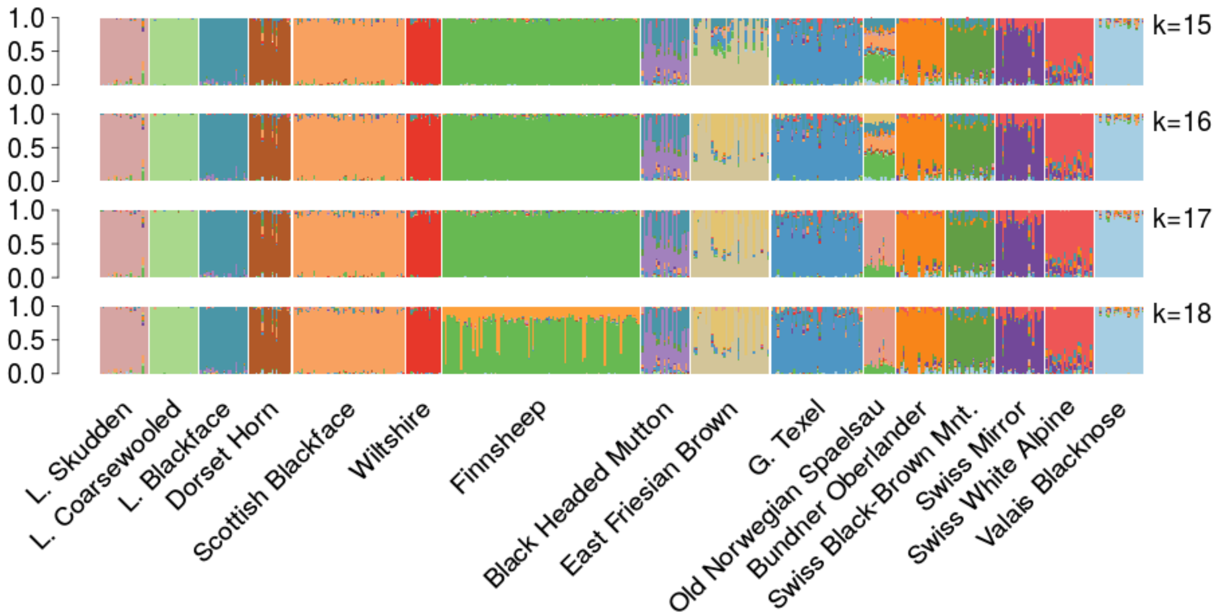

Figure S3. Model based clustering results for k values 15 to 18. The model k = 17 had the highest loglikelihood among the tested models.
